# Fluctuating insect diversity, abundance and biomass across agricultural landscapes

**DOI:** 10.1038/s41598-022-20989-9

**Published:** 2022-10-21

**Authors:** Axel Hausmann, Werner Ulrich, Andreas H. Segerer, Thomas Greifenstein, Johannes Knubben, Jerôme Morinière, Vedran Bozicevic, Dieter Doczkal, Armin Günter, Jörg Müller, Jan Christian Habel

**Affiliations:** 1 Bavarian Natural History Collections (SNSB-ZSM), 81247 Munich, Germany; 2 Department of Ecology and Biogeography, Nicolaus Copernicus University Toruń, 87100 Toruń, Poland; 3 HIPP, 85276 Pfaffenhofen (Ilm), Germany; 4 Advanced Identification Methods GmbH (AIM), 04179 Lepizig, Germany; 5 Field Station Fabrikschleichach, Department of Animal Ecology and Tropical Biology, Julius-Maximilians-University Würzburg, Rauhenebrach, Germany; 6 Bavarian Forest National Park, Grafenau, Germany; 7 Evolutionary Zoology, Department of Environment and Biodiversity, University of Salzburg, 5020 Salzburg, Austria

**Keywords:** Biological techniques, Ecology, Genetics, Zoology, Ecology

## Abstract

Habitat destruction and deterioration of habitat quality caused a severe decline of biodiversity, such as insect diversity. In this study, we analyze insect diversity and biomass across agro-environments. We collected flying insects with 20 malaise traps across a landscape mosaic consisting of organic (eight traps) and conventional (four traps) farmland, as well as across agricultural land that has been recently converted from conventional to organic farming (eight traps). Sampling was conducted over 2 years, in 2019 and 2020, with in total 340 sampling events. We measured the dry weight of the captured organisms and identified species diversity by analyzing Operational Taxonomic Units (OTUs) and Barcode Index Numbers (BINs) via metabarcoding. The results obtained show temporal dynamics. The number of OTUs were always higher than the number of BINs. OTUs and BINs were moderately to highly correlated, while the number of OTUs and BINs were only moderately positively correlated with dry biomass. OTUs and BINs as well as biomass were highest in the recently transformed farmland if compared with pure organic and conventional farmland sites, which showed no significant differences in respect of insect diversity. OTU and BIN numbers but not the OTU/BIN ratio significantly decreased with increasing distance from the nearest forest fringe. The numbers of OTUs, BINs and the OTU/BIN proportion, as well as OTU and BIN/biomass proportions varied strongly over seasons, irrespective of agricultural practice. Based on our findings, we suggest to combine data on insect species richness and biomass measured over a period of time, to derive a largely complete and meaningful assessment of biodiversity for a specific region.

## Introduction

Habitat destruction and the deterioration of habitat quality are the main drivers causing worldwide biodiversity loss^1^. Hereby, the transformation of natural ecosystems into agricultural fields, pastures and plantations caused severe losses of natural and extensively used semi-natural habitats, with negative effects on biota. Central Europe suffers particularly under ongoing agricultural intensification and subsequent landscape homogenization, as well as under the abandonment of former extensively used land^2^. These trends leads to the vanishing of heterogeneous and species rich ecosystems, which provide important habitats to many species. In consequence, most species groups are suffering by a reduction of species richness^3^, species abundance^4^, biomass of flying invertebrates^5^, and qualitative changes of species community structure^6^. These changes subsequently impact various interactions, such as plant–insect interactions^7^ as well as insect-animal relationships^8,9^.

Various studies documented the gradual loss of species richness^10^. Arthropods suffer particularly under the intensification of agricultural land-use. Studies have shown that loss of once extensively used habitats leads to the fragmentation of the remaining habitats, most of which are small, geographically isolated and provide only limited long-term habitats and resources for many species^11,12^. In addition, habitat quality suffers strongly from various anthropogenic activities, such as nitrogen loads^13–15^. In addition, the influx of various toxic substances such as pesticides have detrimental effects to the quality of habitats and have lethal effects on many plants and animal species^16,17^. These multiple drivers lead to the vanishing of local populations and thus to significant reductions of species richness, abundances and biomass of arthropods, as recently reported^18^.

Most of the studies on insect diversity examined one a single measure, such as biomass or proxies expressing the level of diversity, such as species richness^4,19^. However, modern sampling methods and subsequent analyses, such as metabarcoding allow a standardized and simultaneous collection of various data to evaluate e.g. species richness, abundance, and biomass. The revolution in DNA sequencing enables the molecular detection of single species and its abundances from the captured biomass of flying insects^20,21^. Thus, extensive data series can be collected easily from various sampling sites in parallel, with standardized collecting methods, and over several months and years.

In this study, we analyze species richness, abundance and biomass of arthropods. We collected flying insects with 20 malaise traps set across a heterogeneous agricultural landscape during the years 2019 and 2020. Sampling was conducted across organically and conventionally farmed land, as well as at sites, which have been recently converted from conventional to organic treatment. The material collected was dried and weighted, and subsequently analyzed using the metabarcoding technique. For the DNA sequences obtained, we calculated Operational Taxonomic Units (OTUs) and Barcode Index Numbers (BINs). Based on these data we will answer the following research questions:

1. Do values and relationships vary among OTUs, BINs and biomass?
2. Do values and relationships between diversity and biomass change over months and seasons?
3. Do environmental conditions such as agricultural treatment and local conditions impact arthropod diversity and biomass?
4. What can we conclude from our results obtained for the development of future biodiversity monitoring schemes?

## Results

**Figure 1:**
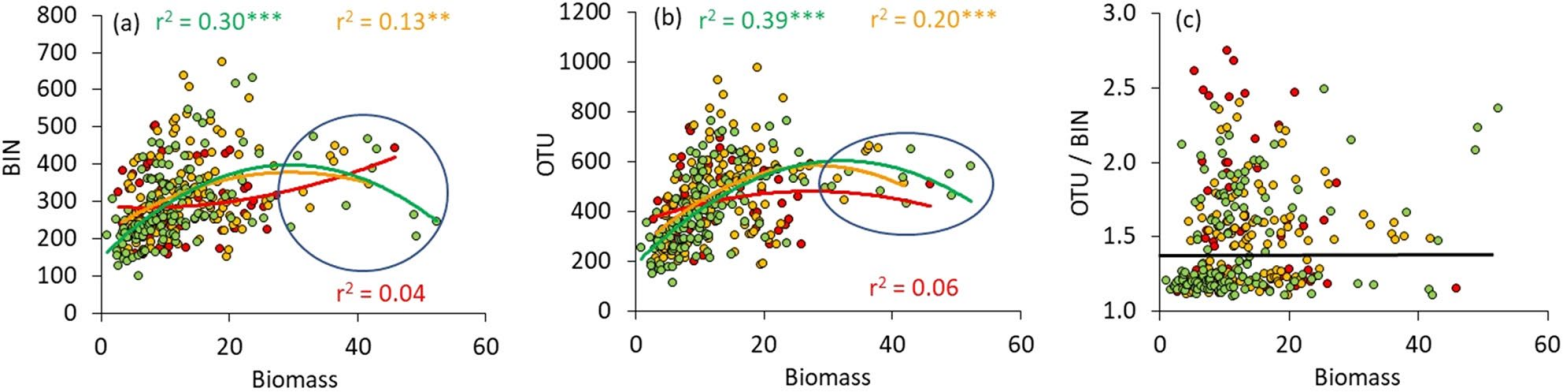
Relationships between numbers of BINs (**a**), OTUs (**b**), OUT/BIN (**c**) and total biomass. Green data points: organic farming, orange: recent change from conventional to organic farming, red: conventional farming. Quadratic least squares regression lines in (**a**,**b**) (with respective coefficients of determination r^2^) peak between 25 and 28 g (except for BINS of conventional farming in **a**). Above the 25 g threshold numbers of BINs and OTUs tend to decrease with increasing biomass (marked by ovals). The black line in (**c**) divides data mainly from 2019 (above) and those obtained in 2020 (below).

In each trap, we found at least 800 different OTUs per year (Appendix 1). In each single sampling event, the numbers of BINs and OTUs exceeded 100 (Fig. 1a,b, Appendix 2: Fig. 1). Both, numbers of OTUs and BINs as well as biomass were highest in the recently transformed farmland sites (Table 2), although these differences among different agricultural practices were not significant after accounting for covariates (Table 3). The number of OTUs were always higher than the number of BINs (Fig. 1c), and both proxis were moderately to highly correlated (2019: *r* = 0.48; *P* < 0.001; 2020: *r* = 0.87, *P* < 0.001). We found marked differences in the OTU/BIN proportion between the two study years (Fig. 1c, Tables 1, 2). Average OTU/BIN proportions were significantly higher in 2019 than in 2020 (Fig. 1c, Appendix 2: Fig. 1).

**Table 1:** Overview of all sampling sites.

Site	Abbrev	Distance to forest	Lat	Long
E-Hof	E1	0	48.5073° N	11.5401° E
E-Hof	E2	15	48.5077° N	11.5400° E
E-Hof	E3	0	48.5043° N	11.5397° E
E-Hof	E4	15	48.5045° N	11.5399° E
E-Hof	E5	50	48.5040° N	11.5402° E
E-Hof	E6	110	48.5032° N	11.5399° E
E-Hof	E7	100	48.5029° N	11.5403° E
E-Hof	E8	80	48.5028° N	11.5402° E
Hagl-Hof	H1	0	48.5091° N	11.5317° E
Hagl-Hof	H2	15	48.5090° N	11.5314° E
Hagl-Hof	H3	0	48.5080° N	11.5294° E
Hagl-Hof	H4	15	48.5079° N	11.5295° E
Hagl-Hof	H5	40	48.5079° N	11.5298° E
Hagl-Hof	H6	130	48.5063° N	11.5319° E
Hagl-Hof	H7	110	48.5063° N	11.5321° E
Hagl-Hof	H8	70	48.5063° N	11.5326° E
Reim-Hof	R1	0	48.4847° N	11.5449° E
Reim-Hof	R2	15	48.4835° N	11.5481° E
Reim-Hof	R3	100	48.4939° N	11.5354° E
Reim-Hof	R4	100	48.4938° N	11.5353° E

The corresponding farm, the abbreviation for the respective sampling point, distance to the forest edge, and the exact GPS coordinates are given.

**Table 2:** Summary data on numbers and relationships of BINs, OTUs, and biomass (in grams).

Year	N	Agricultural practice	OTU	BINs	OTUs/BINs	Biomass	OTU/biomass	BIN/biomass
2019	80	Organic	402 ± 15	255 ± 7	1.58 ± 0.04	13.16 ± 1.16	40.2 ± 2.0	27.0 ± 1.5
2019	80	Conv ⟶ Org	448 ± 16	287 ± 7	1.55 ± 0.04	12.84 ± 0.92	43.6 ± 2.1	30.0 ± 1.8
2019	40	Conventional	414 ± 17	259 ± 10	1.68 ± 0.09	11.49 ± 1.13	48.4 ± 4.9	32.9 ± 4.0
2020	56	Organic	428 ± 22	343 ± 16	1.23 ± 0.02	12.12 ± 1.19	51.1 ± 5.7	41.5 ± 4.8
2020	56	Conv ⟶ Org	487 ± 27	360 ± 18	1.33 ± 0.02	14.47 ± 0.93	39.9 ± 2.8	29.4 ± 1.9
2020	28	Conventional	454 ± 29	338 ± 18	1.33 ± 0.03	13.52 ± 1.59	42.7 ± 4.4	31.9 ± 3.1

*N *number of samples. Errors refer to standard errors.

**Figure 2:**
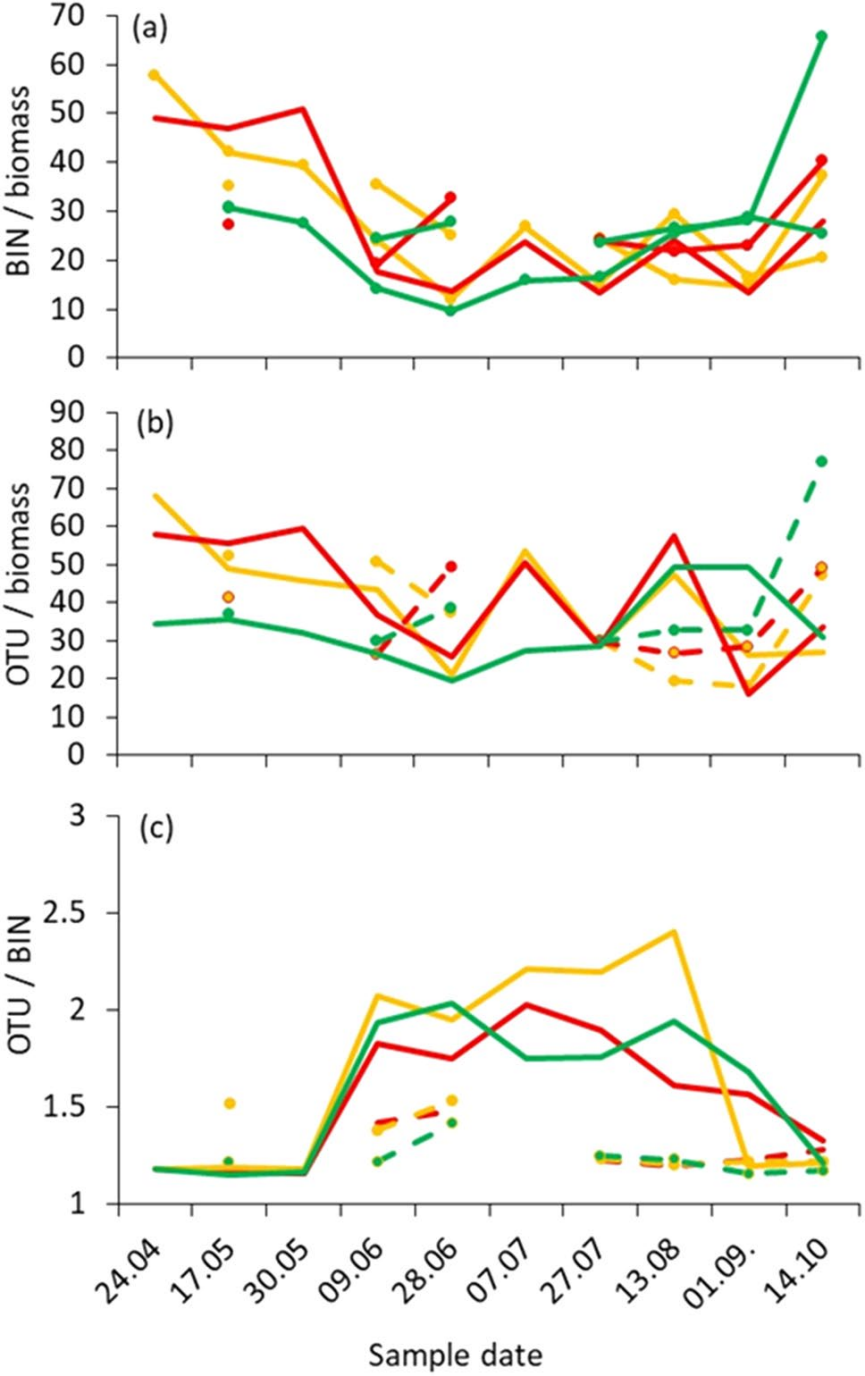
Annual time series of the BIN/biomass (**a**), OTU/biomass (**b**) and OTU/BIN (**c**) relationships for biological farming (green), recent change from conventional to organic farming (orange), and conventional farming (red). Continuous lines: 2019 sampling, broken lines: 2020 sampling.

The numbers of OTUs and BINs were only moderately positively correlated with dry biomass (Fig. 1, Table 3). These correlations were highest for the organic and insignificant for the conventional farmland sites (Fig. 1a,b). OUT and BIN numbers peaked between 25 and 28 g dry biomass (Fig. 1a,b). Only in one sample from the conventional farmland sites we found a total dry biomass > 30 g (Fig. 1a,b). Furthermore, OTU and BIN numbers, but not the OTU/BIN ratio, significantly decreased with increasing distance to the forest edge (Table 3, Appendix 2: Fig. 2a–c).

**Table 3:** Nested general linear modelling (study year nested within agricultural practice) identified major drivers in the number of BINs and OTUs.

Variable	df	BIN		OTU		OTU/BIN		BIN/biomass		OTU/biomass	
		partial η ^2^	β -value	partial η ^2^	β -value	partial η ^2^	β -value	partial η ^2^	β -value	partial η ^2^	β -value
Study year	3	0.17***	–	0.03**	–	0.12***	–	< 0.01	–	< 0.01	–
Agricultural practice	2	0.01	–	0.02	–	0.01	–	< 0.01	–	0.01	–
Distance to woody plants	1	0.11***	− 0.36	0.05***	− 0.26	< 0.01	0.09	< 0.01	< 0.01	< 0.01	0.05
Sample day	1	0.04***	− 0.21	0.02**	− 0.12	< 0.01	0.09	0.01*	− 0.15	< 0.01	− 0.12
(Sample day)^2^	1	0.02**	− 0.19	0.11***	− 0.41	0.10***	− 0.43	0.10***	0.41	0.04***	0.27
ln-biomass	1	0.02**	0.19	0.03***	0.21	< 0.01	0.07	–	–	–	–
r^2^		0.46***		0.43***		0.38***		0.21***		0.09***	

Given are partial η ^2^- and β -values and the coefficient of determination r^2^ of the whole model. N = 340. *df* degrees of freedom. Parametric significances: *P < 0.05, **P < 0.01, ***P < 0.001.

The BIN/biomass (Fig. 2a) and OTU/biomass (Fig. 2b) relationship was generally lowest in the organic farming sites during spring and summer, although we observed strong annual variation in these proportions (Fig. 1). Numbers of OTUs, BINs and the OTU/BIN proportion varied strongly between the seasons and were highest during the summer months, irrespective of agricultural practice (Fig. 2c, Table 3: highly significant squared sampling date factor).

## Discussion

### OTUs, BINs, biomass

We found differences between quantitative and qualitative data collected over time. The number of OTUs was always higher than the number of BINs, as expected. Both proxies were correlated, while the numbers of OTUs and BINs were only moderately positively correlated with dry biomass. We have to consider various limitations when interpreting and comparing trends based on OTUs, BINs and biomass. OTUs frequently produce significant overestimates of species numbers if compared with BINs because many intraspecific genetic polymorphisms might come into play. The algorithm used for BINs is much more realistic in terms of species numbers^22,23^. But of course, we can only detect what is already represented by a haplotype sequence representative in the reference library (in this case BOLD) in order to acquire a BIN. The value of unambiguously assigned BINs essentially depends on how many species are given in the reference library and can be recognized^24–29^. For example, in the BIN analyses, Diptera and Hymenoptera are usually underrepresented (which might underestimate the total number of species assessed, but only if we include BINs > 97%). In our study, the 2019 and 2020 data analyses were both based on the reference libraries of BOLD and GenBank, and a RDP classifier trained on CO1 data^30^. At times, OTUs are recovered there in only one of the two libraries and a classifier. Therefore, a “consensus taxonomy” was applied from combined results. We identified only few differences between BOLD and GenBank, mainly due to misidentifications on GenBank (as the BOLD taxonomy of the German fauna is on a very good level after GBOL projects), and because Genbank (and the classifier) do not account for BIN sharing species, such as BOLD.

In addition to these challenges, individual species representing a mass occurrence, and species which are underrepresented in the DNA reference library (“dark taxa”, often belonging to smaller dipterans and hymenopterans) will create non-realistic OTU/BIN proportions. During years with only low levels of insect abundances due to unfavorable weather conditions for most arthropods, many rare species should be cut away from the low end of the abundance distribution, and the OTU/BIN proportion should be lower. However, in 2019, a year with unfavorable environmental conditions for most insects compared with the year 2020, the OTU/BIN proportion was higher. It seems important to consider that the OTU/BIN proportion, i.e. the rate of hits in the genetic reference library strongly depends on the completeness of the library and the analytical settings.

### Temporal dynamics

Our results show a typical progression of biomass and species diversity development over time, with a rapid build-up of biomass and diversity during spring, followed by a gradual leveling off over late summer, and fall towards autumn (Fig. 2). Similar seasonal trends in biomass and diversity of insects have been documented in other studies^31^. Our results show that BINs, OTUs, and biomass vary greatly, spatially as well as temporally, across years as well as over short periods of time (accounting for individual collection events). These fluctuations are very evident for the 2 years of observation, but significant fluctuations also occur within a study year (Table 2, Fig. 2). Fluctuations in insect populations can be very strong and mostly depend on weather conditions and the densities of parasites and predators. Studies on ground beetles have shown that local populations of invertebrates can fluctuate by up to three orders of magnitude^19^. This could result in it being extremely difficult to detect specific species during short monitoring periods, and thus the validity of assessments made at a particular time is highly questionable^32^.

Our results also demonstrate a significant divergence in the temporal trajectories of biomass and species diversity (Fig. 1c). Also conspicuous are strong outliers of certain parameters (here biomass) at certain points in time. A closer examination of the collected insect individuals evidenced that these are mainly some few heavy and highly mobile nocturnal lepidopteran or dipteran species, which had flown into the trap in larger quantities at the corresponding time and thereby significantly increased the biomass, but did not lead to an increase in species diversity (AH, unpublished data). An example is *Autographa gamma*, a migratory moth species which together with the moth species *Luperina testacea* and *Triodia sylvina* on September 13, 2020, in the “HaglHof 7” Malaise trap were responsible for more than 50% of the NGS reads and caused the lowest measured diversity value (BINs/biomass) of all 340 samples (AH, unpublished data). In many other cases, such incongruences between biomass and diversity were correlated, with extreme amounts of NGS-reads for comparatively heavy and/or invasive species like *Delia platura* (Anthomyiidae), *Botanophila fugax* (Anthomyiidae), *Triodia sylvina* (Hepialidae), *Chrysotus cilipes* (Dolichopodidae) and others, apparently occurring in massive abundance. Such events have to be taken into account when applying automatic insect monitoring and when using biomass as an indicator of biodiversity.

### Spatial heterogeneity

Our results show that different agricultural management had no significant effect on invertebrate biomass and diversity in the study years 2019–2020. This seems to contradict other comparative studies analysing the effects of agricultural management on biodiversity^18,33–35^. In general, biodiversity (such as species number, abundance and biomass, as well as functions) is significantly higher on land that is managed by organic farming. And in fact, a clear difference in biodiversity was also recorded for our study area in a previous study comparing conventional agriculture and organic agriculture, with 80% more biomass of flyable arthropods, and about 50% more species diversity of flyable arthropods^21^. This lack of a potential effects from land management on biodiversity in our study could be due to the strong landscape heterogeneity and the mosaic of fields and grasslands treated organically and conventionally. Thus, potential effects of each land use type could become blurred by negative edge effects from conventionally farmed land, as well as positive spill-over effects from organic farmland. In addition, the flight-capable insects surveyed by the malaise traps are highly dispersive and thus potential local effects might become blurred due to the fact of the high nobility of insects and subsequent intermixing of individuals across mosaicking landscapes. Landscape configuration (e.g. field size) might be of even higher relevance for biodiversity than the degree of agricultural management intensity^36^. Our data and results extend a strong correlation between BINs and OTUs on organic farmland, while this relationship is less pronounced on conventionally farmed land. This animates that in conventional farming, biomass usually consists of only a few species.

Some of the study areas were only recently converted from conventional to organic farming. Here, an increase in diversity and biomass was shown for both species diversity and biomass. This positive development occurred immediately and without any time delay (time lacks frequently apply to ecosystem dynamics^37^). However, it can be assumed that the colonization of ecologically demanding and rare species takes much longer, as these species are often sedentary and do not colonize newly created habitats very quickly^38^. For application-oriented nature conservation, this means that the time factor must be taken into account. Therefore, flowering areas should be maintained as such for as long as possible and not be plowed up again after only a few years, since rare species usually only settle after a few years—at a time when most newly created habitats are destroyed again.

While the type of agricultural use showed minor effects on biomass and diversity of invertebrates, habitat structures in the immediate vicinity of the sampling sites show a large effect. The greatest biomass and diversity was measured at the edge of forest, while comparably low values were obtained in the middle of meadows. Numerous studies have already demonstrated that the immediate supply of ecological niches provided by adjacent habitat diversity has a large effect on species diversity^36,39^. Transitions between open land and forest provide valuable transitional habitats for species from both open land and forest^40,41^. In addition, many flying insects disperse along linear structures, such as forest fringes, and thus accumulate there—and in malaise traps set close to the forest edge^42^.

## Conclusion

Our data show that OTUs, BINs and biomass correlate only to a limited extent, and that local as well as temporal variations are very common. Therefore, it is essential to record different parameters in the field in parallel and over a certain period of time^21,42^. In order to conduct biodiversity monitoring on a large spatial scale, metabarcoding approach offers the basic prerequisite for processing large collections. However, it must be kept in mind that this approach can only provide limited information about the abundance of individual species. And, species community analysis can be invented using metabarcoding data based on presence-absence information of species only to a limited extent; the abundance of individual species is a crucial value for making statements about the structure of a species community. Therefore, aside of metabarcoding, also classical collections for meaningful species groups as well as for problematic groups for DNA barcoding (such as Syrphidae^28^) needs to be carried out. Hereby, not exclusively ‘aerial plankton’ (which by nature already moves over sometimes large spatial scales and thus does not represent potential effects of local management practices very well), but also less dispersive species groups (such as soil-and ground-dwelling fauna) should be considered.

## Material and methods

### Study sites

**Figure 3:**
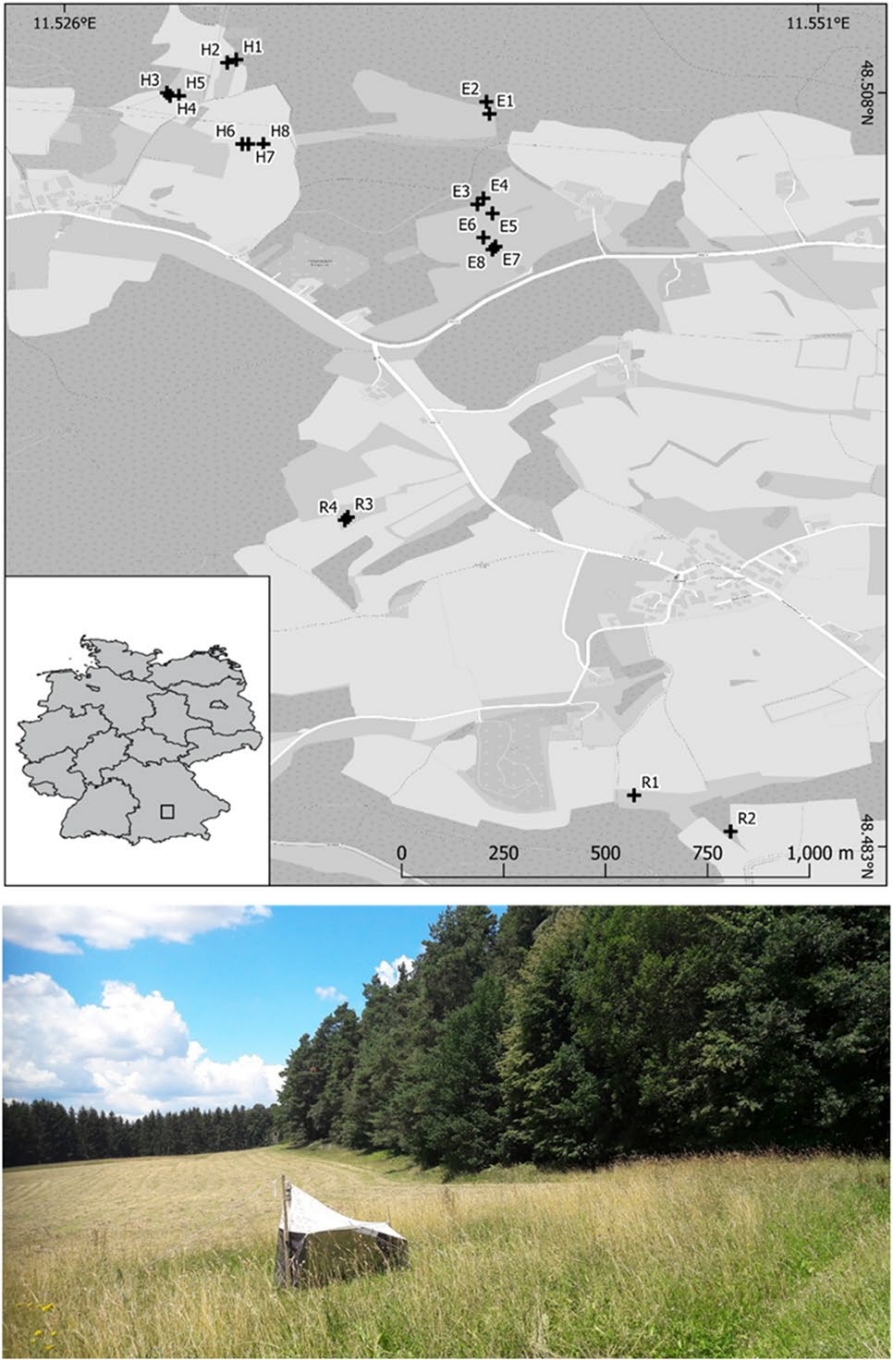
Study area in southern Germany (small inlet map), and locations of the 20 Malaise traps set across the agricultural landscape. Indicated are agricultural fields (light grey), meadows (grey), forest (dark grey), and settlement area. One of our Malaise traps is shown on the picture below. The map was reproduced from the OpenTopoMap web site (https://opentopomap.org) under creative common licence CC BY-SA 3.0 (https://creativecommons.org/licenses/by-sa/3.0/legalcode), with subsequent highlighting of the study area. Photographs were taken by AHS.

Our study area is located in southern Germany, 15 km distant to the city Pfaffenhofen. This study area is characterized by comparatively high topographical heterogeneity. The study region is located in the tertiary hill country, on the edge of the Hallertau region. The soils are mostly deep and fertile and therefore the soil fertility is high. The climate is generally warm to temperate (annual average 9.6 °C). There is significant precipitation throughout the year (with a total precipitation of 943 mm) (https://de.climate-data.org/europa/deutschland). The farms with their cultivated areas are interwoven with each other and represent agricultural fields, grasslands, forest and settlements (see Fig. 3). Conventional and organic agriculture are not clearly spatially separated from each other, as the fields of the different farms form a mosaic. The organic farm conducts extensive cow farming and grasslands. Organic farmland are mowed twice a year and without any application of pesticides, however with the use of organic fertilizers. The conventionally managed farmland belongs to a dairy farm. On these sites, mainly hay as well as silage and hops are cultivated. Conventional farmland are mowed several times (> 2) a year and treated with artificial fertilizers. Pesticides are applied in the conventional farmland area (Broadway (130 g 0.5 L/ha; 14.4.2018), Gardo Gold (3 L/ha; 27.5.2018), Callisto (0.75 L/ha; 27.5.2018) and the shortcut Chlormequat (0.3 L/ha; 12.5.2018)). The farmland which has been recently converted from conventional into organic agriculture produces hay. We established twenty traps in a landscape mosaic consisting of organic (8 traps) and conventional (4 traps) farmland and in farmland that has been recently converted from conventional into organic farming (8 traps). Distances among traps were at least 200 m to minimize potential effects from spatial autocorrelation. Some of the traps were set in the center of a meadow, others close to the forest fringe. Details of each single sampling site (Malaise trap) are given in Table 1.

### Malaise traps

We installed 20 standard Malaise traps (height front 180 cm, height rear 120 cm, length 180 cm, width front and rear 110 cm) (B&S Entomological services). All 20 traps were activated from April till October during the years 2019 and 2020. All traps were southwards positioned and with similar exposure to wind. The traps were activated simultaneously. 600 ml sampling bottles were filled with 80% ethanol. All Malaise traps were emptied simultaneously to guarantee comparability every 9–36 days (mean 23.3 days) (exact dates are given in Appendix 1), resulting in a total of 340 single samples. The material was stored in pure alcohol until DNA sequencing. A complete list of raw data are given in Appendix 1.

### Biomass

Dry and wet biomass material was weighted and analyzed separately, according to Ssymank et al.^42^. Species were dried according to size selection using a sieve (in diameter: 6.5 mm) in diameter in a 70 °C oven over night (or at least for 8 h).

### Metabarcoding

After drying the organic material of the Malaise traps, species identification was performed using DNA metabarcoding following the methodology described in Hausmann et al.^21^. Complete drying of the material is essential for the elimination of ethanol and successful molecular genetic processing^29,43–46^. Each single dried sample (altogether 340 samples) was homogenized in a FastPrep96 machine (MP Biomedicals) using sterile steal beads in order to generate a homogeneous mixture of arthropods. Homogenized tissue samples were subsequently sent out for metabarcoding (conducted by AIM GmbH, Leipzig, Germany). Prior to DNA extraction, 1 mg of each homogenisate was weighed into sample vials and processed using adapted volumes of lysis buffer with the DNeasy 96 blood & tissue kit (Qiagen, Hilden, Germany) according to the manufacturer’s protocol. The mitochondrial DNA barcode CO1-5P target region was amplified using a 313 bp long mini-barcode by PCR^47,48^, using forward and reverse HTS primers, equipped with complementary sites for the Illumina sequencing tails. In a subsequent PCR reaction, index primers with unique i5 and i7 inline tags and sequencing tails were used for amplification of indexed amplicons. Afterwards, equimolar amplicon pools were created and size selected using preparative gel electrophoresis.

DNA concentrations were measured using a Qubit fluorometer, and adjusted to 40 µl pools containing equimolar concentrations of 100 ng DNA template each. Pools were purified using MagSi-NGSprep Plus (Steinbrenner Laborsysteme GmbH, Wiesenbach, Germany) beads. A final elution volume of 20 µl was used. High Throughput Sequencing (HTS) was performed on an Illumina MiSeq (Illumina Inc., San Diego, USA) using v3 chemistry (2 × 300 basepairs, 600 cycles, maximum of 25 million paired-end reads).

Raw FASTQ files from Illumina were bioinformatically pre-processed using VSEARCH v2.9.1^49,50^, and as described in more detail in Hausmann et al.^21^. Briefly, paired-end reads were merged and forward and reverse adapter sequences removed from each read. Reads that did not contain the appropriate adapter sequences were discarded. The resulting reads were dereplicated and those of short length and/or low quality were filtered out. Chimeric sequences were removed using the de novo algorithm. Finally, reads were clustered into OTUs using global pairwise alignment followed by de novo distance-based greedy clustering (at 98% pairwise identity) to the closest centroid sequence. Centroids, defined initially as the most abundant reads at the level of the entire dataset, were kept as the representatives of OTUs, and the resulting OTU FASTA file used as a reference database to create an OTU table of read counts in each sample. OTUs were blasted using Geneious (v.10.2.5—Biomatters, Auckland—New Zealand) against (1) a custom, taxonomically annotated Animalia database downloaded from BOLD^48^ and (2) a local copy of the NCBI nucleotide database downloaded from ftp://ftp.ncbi.nlm.nih.gov/blast/db/(both downloaded on September 25, 2020).

Top BLAST hits for each OTU were exported from Geneious, combined with the OTU table produced by the pre-processing pipeline, and noise-filtered as described in Hausmann et al., 2020^21^. Interactive Krona charts were produced from the taxonomic information using KronaTools v1.3^51^.

Species identification in the Malaise trap samples was based on High Throughput Sequencing (HTS) data grouped to genetic clusters (OTUs), blasted and assigned to barcode index numbers (‘BINs’: Ratnasingham and Hebert^22^) which are considered to be a good proxy for species numbers^22,23^. In our case, the detailed analysis of the Lepidoptera data revealed that the frequency of ‘false positives’ (0.5%) and BIN-sharing (1.5%) obstructing species discrimination (but nevertheless still pointing to species complexes) played a negligible role (see results for details).

### Statistics

For each of the 340 Malaise trap sample data we calculated the OTU/biomass, BIN/biomass and OTU/BIN relationships. We used ordinary parametric least squares regression and parametric nested general linear modelling (as implemented in Statistica 12.0) to relate numbers of OTUs, BINs, OUT/biomass, BIN/biomass, and OTU/BIN (response variables) to study year and agricultural practice (fixed categorical predictors, study year nested within agricultural practice), and to distance from forest edge, sample day, and ln-transformed biomass (metric predictors). As predictors were measured at different units, we report β-values and focus on partial η-square values as measures of effect sizes. Study sites were sampled at different days across the 2 years (not identical days for the years 2019 and 2020). To account for this possible bias, we included sample day and the squared, zero centred sample days (= (day-average sample day)^2^) in the model. Metric predictors were only moderately correlated (r < 0.5). Traps operated at distances of at least 200 m guaranteeing spatial non-independence due to the relatively small sample areas of Malaise traps.

## Supplementary Information

Supplementary Information 1.

Supplementary Information 2.
