# Supplementary material for: Fluctuating insect diversity, abundance and biomass across agricultural landscapes
Source: Sci Rep. 2022 Oct 21;12:17706. doi: 10.1038/s41598-022-20989-9 (PMC9587014; doi:10.1038/s41598-022-20989-9)

**Appendix 2**

Figure 1: Relationships between the number of BINs and OTUs. Green data points: biological farming, red: recent conversion from traditional to biological farming, orange: conventional farming. The black line in divides data mainly from 1919 (below) and those obtained in 2020 (above).


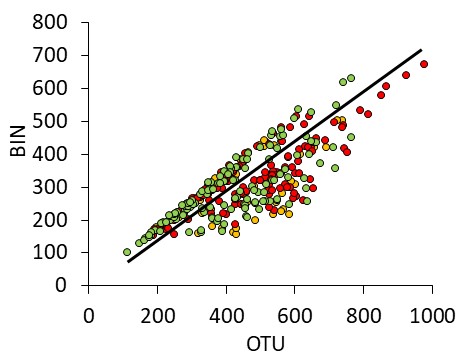


Figure 2: Dependencies of BINs, OTUs, and the OTU / BIN ratio on sampling day and distances to the nearest woody plants. Green data points: biological farming, red: recent conversion from traditional to biological farming, orange: conventional farming. Parametric significances of linear (a-c) and 2^nd^ order polynomic (d-f) ordinary least squares regressions: ***: P < 0.001.


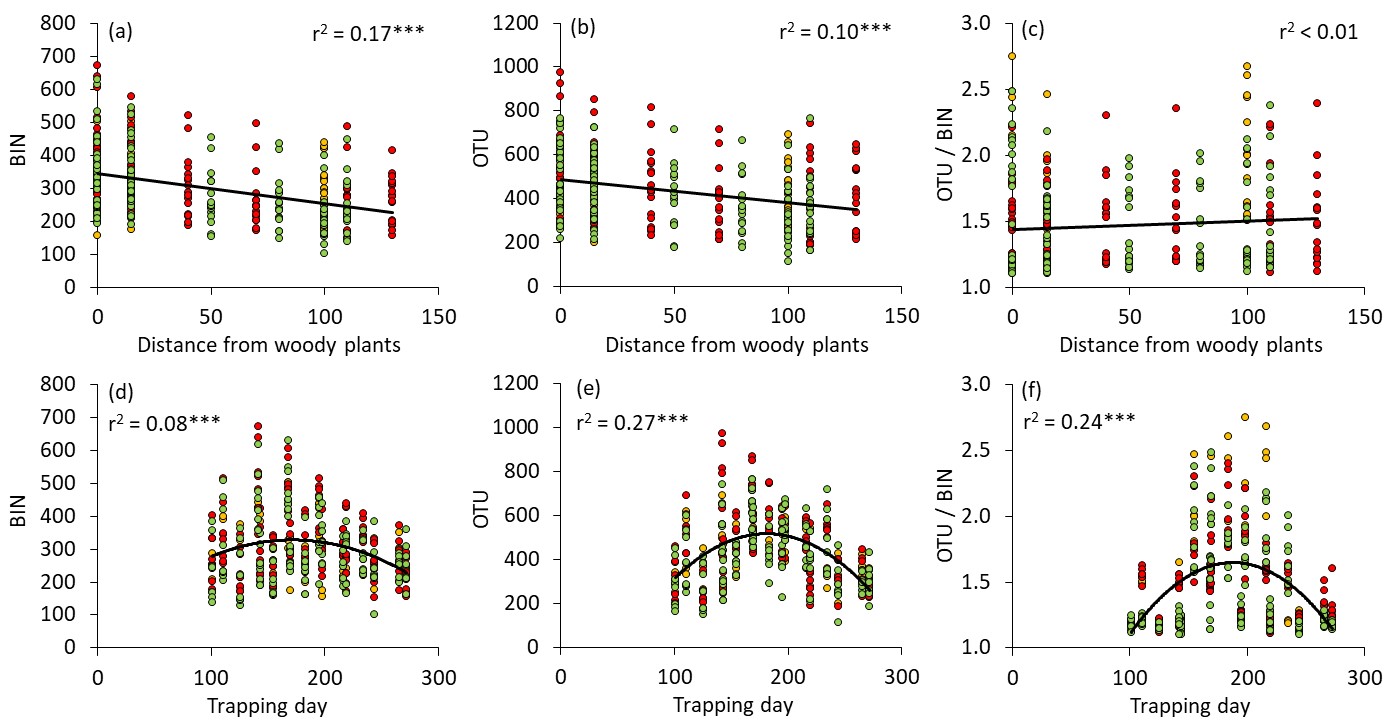


Figure 3: Phenology of the OTU / biomass relationship in 2019 (continuous lines) and 2020 (broken lines).


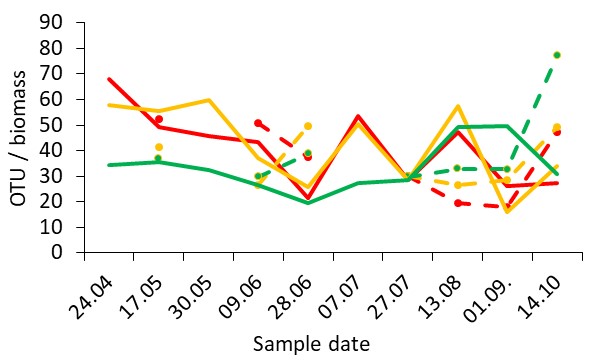

Supplement: Supplementary file 2 — Supplementary Information 2. [file 41598_2022_20989_MOESM2_ESM.docx]
